# Squamous Cell Carcinoma of the Thyroid as a Result of Anaplastic Transformation from BRAF-Positive Papillary Thyroid Cancer

**DOI:** 10.1155/2017/4276435

**Published:** 2017-10-12

**Authors:** Alina Basnet, Aakriti Pandita, Joseph Fullmer, Abirami Sivapiragasam

**Affiliations:** ^1^Department of Hematology Oncology, SUNY Upstate Medical University, Syracuse, NY 13205, USA; ^2^Department of Medicine, SUNY Upstate Medical University, Syracuse, NY 13205, USA; ^3^Department of Pathology, SUNY Upstate Medical University, Syracuse, NY 13205, USA

## Abstract

Papillary thyroid carcinoma (PTC) is the most common malignant neoplasm of the thyroid. Majority of the PTC carries an excellent prognosis. However, patients with tall cell variant (TCV) of papillary thyroid carcinoma have a worse prognosis than those with the classic variant. On the other hand, squamous cell carcinoma of the thyroid (SCT) is an unusual neoplasm thought to arise as a primary tumor or as a component of an anaplastic or undifferentiated carcinoma. We report a patient with TCV of PTC presenting years later with squamous transformation. In addition, the patient was found to have BRAF mutation. Such dedifferentiation is considered to be a rare phenomenon and has been reported only in the form of case reports in the literature. The relationship between BRAFV600E mutation and squamous cell transformation of papillary thyroid cancer is unknown at this time. Meticulous pathology is needed to identify such variants. Our patient responded to treatment with concurrent chemotherapy with carboplatin and paclitaxel along with radiation.

## 1. Introduction

Papillary thyroid carcinoma (PTC) is the most common malignant neoplasm of the thyroid [1]. Tall cell variant (TCV) of PTC was originally defined by Hawk and Hazard, and this group is recognized by cells with height that is at least two times the width [2]. The incidence of papillary thyroid cancer varies between 3.2 and 19% [2–5]. However, patients with TCV of papillary thyroid carcinoma have a worse prognosis than those with the classic variant [6]. On the other hand, squamous cell carcinoma of the thyroid (SCT) is an unusual neoplasm thought to arise as a primary tumor or as a component of an anaplastic or undifferentiated carcinoma [7]. It is often mixed with heterogeneous elements and is usually associated with areas of well-differentiated papillary or follicular carcinoma [7]. We report a patient with TCV of PTC presenting years later with squamous transformation. In addition, the patient was found to have BRAF mutation which confers a very poor prognosis.

## 2. Case Report

**Figure 1:**
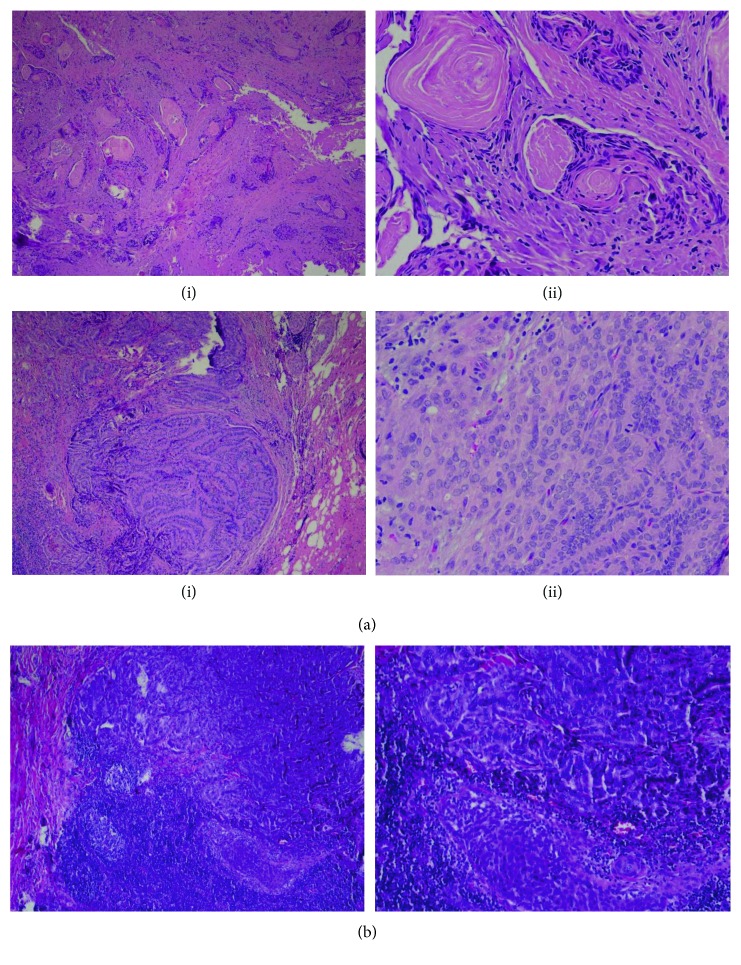
(a) H&E staining of the thyroid bed and paratracheal lymph node. Top row, from left to right: (i) squamous cell carcinoma in the thyroid bed; (ii) keratin pearls of squamous cell carcinoma in higher power. Bottom row, from left to right: (i) paratracheal lymph node showing papillary feature of thyroid cancer; (ii) tall cell areas showing mitotic figure in higher power. (b) From left to right, low power showing papillary thyroid cancer and squamous cell cancer in the same field adjacent to each other.

**Figure 2:**
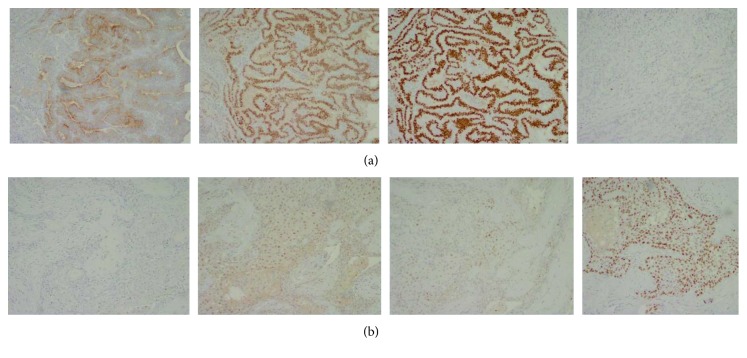
(a) From left to right are the papillary tall cell variant portion of thyroid cancer staining positive for thyroglobulin, PAX8, and TTF-1 and negative for p63. (b) From left to right are squamous cell area of thyroid cancer staining negative for thyroglobulin, weakly positive for PAX8 and TTF-1, and strongly positive for p63.

Our patient is a 56-year-old male who was diagnosed with TCV of PTC in 2011 when he presented with multiple enlarged cervical lymph nodes. He underwent total thyroidectomy with central compartment neck dissection followed by radioactive iodine (RAI) therapy. His tumor was pTNM (pathologic primary tumor, regional node, and distant metastasis) pT2N1bM0-stage I. Six months later, he had an early local recurrence in the left neck, for which he underwent a left neck dissection. After about a year, he had a positron emission tomography (PET) scan that showed activity in the right neck, and he underwent a right neck dissection. Then, he was followed up with surveillance scans. Three years later, a PET scan showed uptake suggestive of recurrence along the left thyroid bed as well as activity along the right paratracheal region. Fine-needle aspiration of the left thyroid bed as well as a right paratracheal node came back positive for recurrent papillary thyroid carcinoma. At that time, he was symptomatic with more fatigue and weight loss. He then underwent revision of left paratracheal and central neck dissection with right paratracheal and mediastinal lymph node dissection along with shave biopsy of the right neck lesion. Histopathology of one of the right paratracheal lymph nodes showed metastatic poorly differentiated thyroid carcinoma composed of papillary tall cell phenotype involving one lymph node. In addition, the left thyroid bed and a second right paratracheal lymph node demonstrated squamous cell carcinoma (Figures 1(a) and 1(b)). The poorly differentiated papillary thyroid carcinoma component was positive for TTF-1, thyroglobulin, and PAX8 while the squamous cell carcinoma was positive for p63, PAX8 (focally), and TTF-1 (very focally positive) and negative for thyroglobulin (Figures 2(a) and 2(b)). This pattern of immunohistochemistry suggests that the squamous variant seen was actually a transformation from papillary cancer unlike the primary squamous cancer which stains negative for TTF-1 and PAX8. The specimen also tested positive for BRAF mutation via immunohistochemistry (IHC). We then retrospectively analyzed his primary tumor for BRAF, and his primary tumor was positive for BRAF as well. He was started on concurrent radiation and chemotherapy with weekly dosing of carboplatin and Taxol. He went on to complete that over the course of 6 weeks. PET-CT was done after 12 weeks of completion of therapy and suggested near-complete resolution of metabolic activity in the thyroid bed and regional lymph node areas.

## 3. Discussion

Several investigators found that the TCV of PTC was more aggressive than ordinary well-differentiated papillary carcinoma [8]. Tall cell variant tends to have more frequent extrathyroidal extension with higher recurrence and mortality rate [9]. Papillary carcinomas are also described with several other variants like focal insular component, spindle and giant cell carcinoma, squamous cell carcinoma, and mucoepidermoid carcinoma [10]. It is not rare to find papillary carcinoma showing characteristics of more than one variant [1]. Squamous transformation, however, is usually rare and has been described only in the form of case reports in the literature [11–13]. It is also suggested that the tall cell variant can evolve into the spindle cell type of squamous cell carcinoma [4, 14]. LiVolsi and Merino suggested that, in most cases, squamous cell carcinomas appear as a result of metaplasia of follicular epithelial cells [15].

Bronner and LiVolsi described five tumors which were composed of squamous cell carcinoma of thyroid (SCT) and TCV [14]. These tumors behaved in an aggressive fashion [14]. LiVolsi and Merino described eight cases of primary SCT with extension in perithyroidal soft tissues of the neck, with prominent vascular invasion (two cases) and perineural invasion (one case) [15]. Kleer et al. found 4 out of 8 cases of SCT to be associated with tall cell variant. p53 expression and high MIB1 labeling index conferred a worse prognosis [7] with frequent capsular and vascular invasion as well as tumor recurrence even after excision.

When histological biopsy from any head and neck yields SCC, it is integral to consider if transformation has occurred [12]. Squamous cells can be found in the thyroid from persistence of thyroglossal ducts or brachial pouch–derived structures or from squamous metaplasia in Hashimoto's thyroiditis. In the presence of squamous cell carcinoma involving the thyroid, direct involvement from the tumor of the larynx or trachea should be ruled out in addition to metastasis from the lungs.

While there is no recognized pattern of progression from differentiated thyroid carcinoma to a particular form of anaplastic carcinoma, Bronner and LiVolsi reported significant risk factors for progression which include preexisting thyroid neoplasm, radiation therapy to the neck region, and I-131 therapy [4, 15].

Due to the rarity of this tumor variant, there is no consensus for its management. A case series has described surgery with re-resections, external beam radiation therapy, and chemotherapy as treatment options [12]. Chemotherapy concurrent with radiation has been described in the literature. We adopted the same approach for our patient, and he tolerated the treatment well.

BRAFV600E mutation has been found positive in the tall cell areas as well as squamous cell transformation, whereas in our patient tall cell portion was positive and squamous cell transformation was only focally positive [16]. Other high-risk factors are male gender, tumor size >5 mm, bilateral/multifocal location, lower third of the thyroid lobe location, lymph node metastasis at presentation, superficial tumor location, capsule invasion/extrathyroidal extension, and stromal fibrosis [10].

Our patient had recurrence with BRAF mutation, which is closely associated with aggressive clinic pathological characteristics that led to poorer outcome in papillary thyroid cancer. Accordingly, aggressive treatment should be considered for papillary thyroid cancer patients with BRAF mutation [17]. A meta-analysis of 20,764 patients suggested that the BRAFV600E mutation is associated with several high-risk clinical variables used in prognostic staging systems, including extrathyroidal invasion, high TNM stage, lymph node metastasis, recurrence, and overall survival. However, distant metastasis occurrence was less in tumors with BRAF mutation. Thus, the overall survival effect of BRAFV600E mutation on PTC tumor remains to be determined.
